# *Fusarium* species isolated from post-hatchling loggerhead sea turtles (*Caretta caretta*) in South Africa

**DOI:** 10.1038/s41598-022-06840-1

**Published:** 2022-04-07

**Authors:** Mariska R. Greeff-Laubscher, Karin Jacobs

**Affiliations:** 1 Water Research Group, Unit Environmental Sciences and Management, Potchefstroom Campus, North-West University, Private Bag X6001, Potchefstroom, 2520 South Africa; 2 Department of Microbiology, Stellenbosch University, Stellenbosch, 7599 South Africa

**Keywords:** Microbiology, Molecular biology, Ecology

## Abstract

Species in the *Fusarium solani* species complex are fast growing, environmental saprophytic fungi. Members of this genus are filamentous fungi with a wide geographical distribution. *Fusarium keratoplasticum* and *F. falciforme* have previously been isolated from sea turtle nests and have been associated with high egg mortality rates. Skin lesions were observed in a number of stranded, post-hatchling loggerhead sea turtles (*Caretta caretta*) in a rehabilitation facility in South Africa. Fungal hyphae were observed in epidermal scrapes of affected turtles and were isolated. The aim of this study was to characterise the *Fusarium* species that were isolated from these post-hatchling loggerhead sea turtles (*Caretta caretta*) that washed up on beaches along the South African coastline. Three gene regions were amplified and sequenced, namely the internal transcribed spacer region (ITS), a part of the nuclear large subunit (LSU), and part of the translation elongation factor 1 α (*tef1*) gene region. Molecular characteristics of strains isolated during this study showed high similarity with *Fusarium* isolates, which have previously been associated with high egg mortality rates in loggerhead sea turtles. This is the first record of *F. keratoplasticum, F. falciforme* and *F. crassum* isolated from stranded post-hatchling loggerhead sea turtles in South Africa.

## Introduction

The ascomycete genus *Fusarium* (*Hypocreales, Nectriaceae*) is widely distributed in nature and can be found in soil, plants and different organic substrates. This genus represents a diverse complex of over 60 phylogenetically distinct species^1–3^. Some species, specifically those forming part of the *Fusarium solani* species complex (FSSC)^4^, are known pathogenic species, and have been associated with human, plant and animal infections—in both immunocompromised and healthy individuals^1,5–8^. Phylogenetically, this group comprises three major clades, of which clade I forms the basal clade to the two sister clades II and III. Members of clade I and II are most often associated with plant infections and consequently have limited geographical distributions^4^. Members of clade III represent the highest phylogenetic and ecological diversity and are most commonly associated with human and animal infections^4^. Species represented in this clade are typically regarded as fast growing and produce large numbers of microconidia. This facilitates distribution within the host and its environment and promotes virulence. Clade III, further consists of three smaller clades, namely clades A, B and C. While clades A (also known as the *F. falciforme* clade) and C (also known as the *F. keratoplasticum* clade) consist predominantly of isolates from humans and animals, plant pathogens constitute most isolates represented in clade B^1,7^.

*Fusarium* spp. have been identified in infections of marine animals including (but not limited to); bonnethead sharks (*Sphyrna tiburo*)^9^, scalloped hammerhead sharks (*Sphyma lewini*)^10^, and black spotted stingray (*Taeniura melanopsila*)^6^. Strains from this genus have been reported to cause skin and systemic infections in marine turtles^5,11–15^, and are considered to be one of many threats to turtle populations worldwide causing egg infections and brood failure in 6 out of seven turtle species^7,16^. Challenge inoculation experiments provided evidence of pathogenicity for *F. keratoplasticum,* a causative agent of sea turtle egg fusariosis (STEF) in loggerhead sea turtle populations in Cape Verde^17^. Since then, *Fusarium* spp.*,* or more specifically *F. falciforme* and *F. keratoplasticum* have increasingly been isolated from turtle eggs and nests. Subsequent research studies have isolated *F. falciforme* and *F. keratoplasticum* from infected eggs in turtle nests on beaches along the Atlantic, Pacific and Indian Oceans, as well as the Mediterranean and Caribbean Sea^15,16,18–23^. Both *F. keratoplasticum* and *F. falciforme* are pathogenic to turtle eggs and embryos, and are able to survive independent of the hosts^7,17^. In recent years, members from *F. falciforme* and *F. keratoplasticum* of clade III, have been described as emerging animal pathogens, causing both localised and systemic infections^6,16,17,23^. These infections can result in mortality rates as high as 80–90% in animal populations^7,17^. Cafarchia and colleagues (2019) suggested that fusariosis should be included in differential diagnosis of shell and skin lesions in sea turtles and that species level identification is required to administer appropriate treatment and infection control^12^.

**Figure 1:**
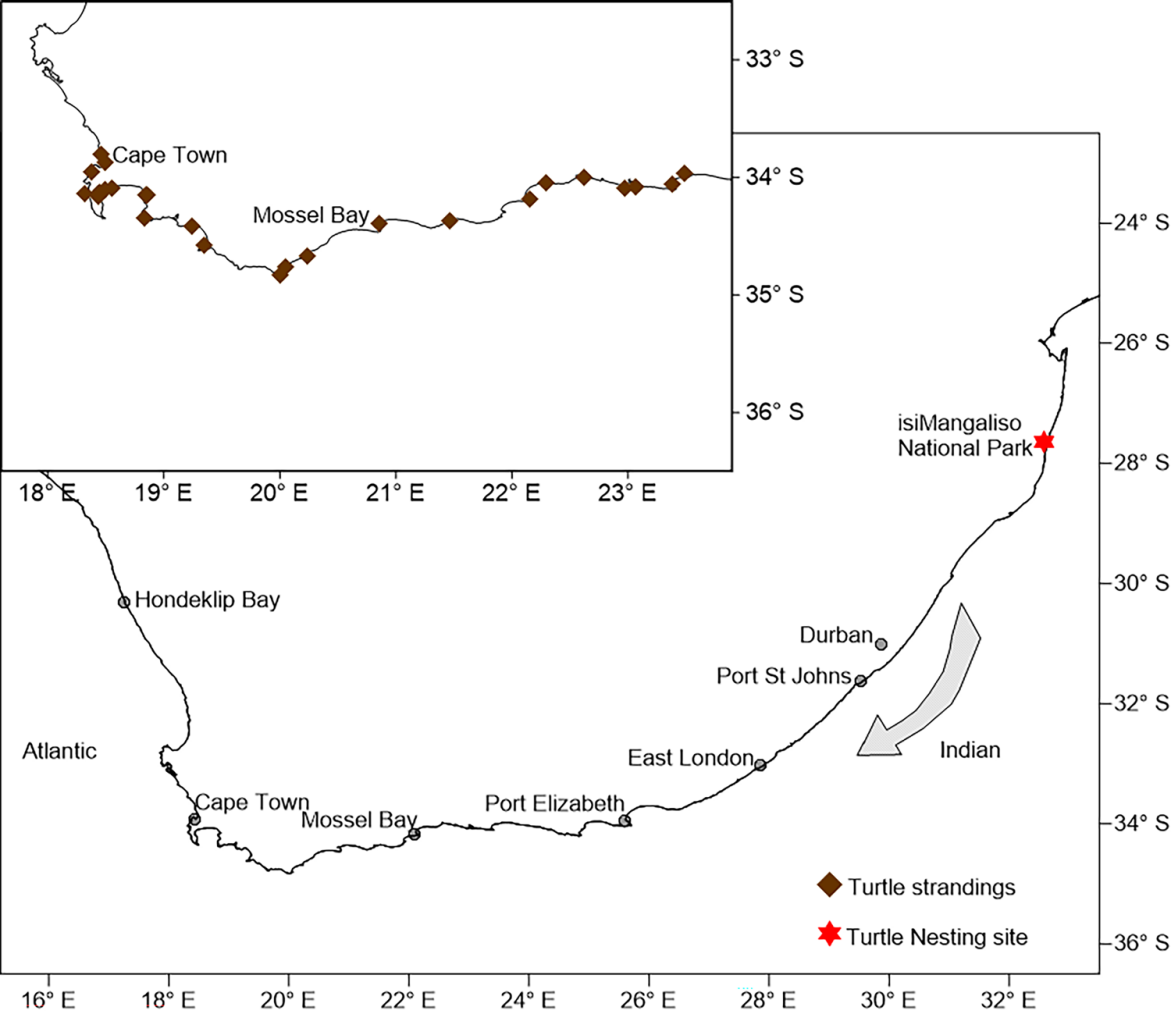
Map showing the South African coastline, indicating nesting sites and sites where post-hatchling sea turtles were found along the coastline between Mossel Bay and False Bay.

Loggerhead sea turtles nest on the beaches of Southern Africa between November and January^24,25^. Hatchlings that find their way into the ocean are carried south in the Aghulas current, with some turtles stranding on the South African coast, mainly between the months of March and May each year. Between 2015 and 2016, a total of 222 post-hatchling (turtles that have absorbed the yolk-sac and are feeding in open ocean but have yet to return to coastal waters to enter the juvenile stage) loggerhead sea turtles were admitted to a rehabilitation centre after stranding along the Indian and Atlantic Ocean coastline of South Africa, between Mossel Bay and False Bay (Fig. 1). During their time at the rehabilitation centre a number of these turtles developed skin lesions. Fungal dermatitis was diagnosed based on skin scrape cytology findings. Fungal strains resembling *Fusarium* were isolated from the affected areas*.*

The aim of this study was to characterise the strains isolated from skin lesions of post-hatchling loggerhead Sea turtles that washed up on beaches along the South African coastline, and to determine the molecular relationships between these isolates and those strains reported from literature that pose significant conservation risks to sea turtles from other geographic localities.

## Materials and methods

### Gross observations and Fungal isolations

Post-hatchling turtles with skin lesions were isolated from unaffected turtles. Clinical signs observed were as follows; excessive epidermal sloughing on the limbs, head and neck, where scales on the skin lifted easily and were frequently lost. A softening and sloughing of the carapace and plastron were observed, where scutes of the carapace and plastron became crumbly, soft and were frequently shed. Turtles were diagnosed with fungal skin infection if they had clinical signs of epidermal sloughing and a positive epidermal scrape. Epidermal scrapes taken from lesions of affected turtles were examined by light microscopy (20 to 50 × objective) and deemed positive if significant numbers of hyphae were observed. For fungal isolation, samples (scrapings) were taken from affected areas of skin in a sterile manner and placed onto culture media. During 2015 and 2016, 10 fungal isolates were isolated from 10 clinically affected loggerhead sea turtles (*Caretta caretta*) onto marine phycomycetes isolation agar (12.0 g Agar, 1.0 g Glucose, 1.0 g Gelatin hydrolysate, 0.01 g Liver extract, 0.1 g Yeast extract, 1 000 mL Sea water) supplemented with streptomycin sulphate and penicillin [0.05% (w/ v)] to prevent bacterial growth^26^. Plates were incubated at 20 °C and monitored daily for fungal growth. Following 3 days of incubation, emerging hyphal tips were aseptically transferred with a sterile needle onto potato dextrose agar (PDA) and incubated. Single spore cultures were obtained by taking a needle tip full of hyphae from a 14 day old culture on PDA, mixing it with 1 mL sterile MilliQ water and spreading 80 µL onto 1.5% water agar plates. Plates were incubated overnight at room temperature. Following incubation, 8 single, germinated microconidia were transferred onto 2 PDA plates (4 microconidia on each plate). After 3 days of incubation at 26 ± 1 °C, all 8 colonies were examined. Colonies with similar colour and hyphal growth were regarded as the same isolate and one colony was selected for characterisation. When differences were observed, one of each different colony was selected for further characterisation. Based on gross observations of single spore colonies 14 distinct isolates were identified for molecular characterisation. Agar plugs (6 mm diameter) of the chosen colonies were transferred onto PDA and incubated at 26 ± 1 °C for 7 days.

### DNA extractions, molecular characterisation, and phylogenetic analyses

Total genomic DNA was extracted from single spore colonies following incubation for 7 days on PDA. A heat lysis DNA extraction protocol was used^27^. Extracted DNA were stored at − 20 °C until needed. Molecular characterisation was performed based on 3 gene regions for 14 strains. The gene regions included internal transcribed spacer region (ITS), a part of the nuclear large subunit (LSU) and partial translation elongation factor 1-α (*tef1*) gene region^28^. PCR reactions were performed in a total volume of 25 µL, containing 100–200 ng genomic DNA. Kapa ReadyMix (Kapa Biosystems; Catalog #KK1006) was used for PCR reactions. Conditions for the PCR amplification were as follows. Initial denaturation at 94 °C for 5 min, followed by 35 cycles at 94 °C for 45 s, 45 s annealing (see Table 1 for specific annealing temperatures) and 72 °C for 1 min, followed by a final extension at 72 °C for 7 min. Purified PCR products were sequenced by using BigDye Terminator Cycle Sequencing Kit (Applied Biosystems) and an ABI PRISM 310 genetic analyser. Sequencing was done in one direction. Each sequence was edited in BioEdit sequence alignment editor v7.2.5. Phylogenetic analyses were conducted using the dataset from Sandoval-Denis et al*.* (2019) combining sequences of three loci (LSU, ITS and *tef1*) to identify species^28–32^ (Table 2 lists all the sequences included in the phylogenetic analyses). Alignments were done in ClustalX using the L-INS-I option. Phylogenetic analysis was performed using Maximum likehood (ML) analysis, with GTR + I + G. The partitioning scheme and substitution models were selected using Partitionfinder v 2.1.1^33^. The software package PAUP was used to construct the phylogenetic trees and confidence was calculated using bootstrap analysis of 1 000 replicates. *Geejayessia atrofusca* was used as an outgroup. A Bayesian analysis was run using MrBayes v. 3.2.6^34^. The analysis included four parallel runs of 500 000 generations, with a sampling frequency of 200 generations. The posterior probability values were calculated after the initial 25% of trees were discarded.

**Table 1:** Primers used for amplification and sequencing.

Primer name	Primer sequence (5′ – 3′)	Annealing temperature (°C)	Reference
ITS 1	TCC GTA GGT GAA CCT GCG G	51.1	^41^
ITS 4	TCC TCC GCT TAT TGA TAT GC		^41^
LSU-00021	ATT ACC CGC TGA ACT TAA GC	63.0	^42^
LSU-1170	GCT ATC CTG AGG GAA ATT TCG G		^43^
EF1	ATG GGT AAG GAR GAC AAG AC	53.6	^31^
EF2	GGA RGT ACC AGT SAT CAT GTT		^31^

**Table 2:** *Fusarium* strains included in the phylogenetic analyses.

Species name	Strain number	Genbank accession number			Source	Origin	Reference
		ITS	LSU	EF			
*Geejayessia atrofusca (outgroup)*	NRRL 22316	AF178423	AF178392	AF178361	*Staphylea trifolia*	USA	^28^
*F. ambrosium*	NRRL 20438	AF178397	DQ236357	AF178332	*Euwallacea fornicatus* on *Camellia sinensis*	India	^28^
	NRRL 22346 = CBS 571.94ET	EU329669	EU329669	FJ240350	*Euwallacea fornicatus* on *Camellia sinensis*	India	^28^
*F. bostrycoides*	CBS 130391	EU329716	EU329716	HM347127	Human eye	Brazil	^28^
	CBS144.25NT	LR583704	LR583912	LR583597	Soil	Honduras	^28^
	NRRL 31169	DQ094396	DQ236438	DQ246923	Human oral wound	USA	^28^
*F. catenatum*	CBS 143229 T = NRRL54993	KC808256	KC808256	KC808214	*Stegostoma fasciatum* multiple tissues	USA	^28^
	NRRL 54992	KC808255	KC808255	KC808213	*Stegostoma fasciatum* multiple tissues	USA	^28^
*F. crassum*	CBS 144386 T	LR583709	LR583917	LR583604	Unknown	France	^28^
	NRRL 46596	GU170647	GU170647	GU170627	Human toenail	Italy	^28^
	NRRL 46703	EU329712	EU329712	HM347126	Nematode egg	Spain	^28^
	ML16006	OM574602	ON237616	ON237630	*Caretta caretta* post-hatchling	South Africa	This study
	ML16011	OM574607	ON237621	ON237635	*Caretta caretta* post-hatchling	South Africa	This study
	ML16012	OM574608	ON237622	ON237636	*Caretta caretta* post-hatchling	South Africa	This study
*F. euwallaceae*	NRRL 54722 = CBS 135854 T	JQ038014	JQ038014	JQ038007	*Euwallacea fornicatus* on *Persea americana*	Israel	^28^
	NRRL 62626	KC691560	KC691560	KC691532	*Euwallacea fornicatus* on *Persea americana*	USA	^28^
*F. falciforme*	033 FUS		KC573932	KC573883	*Chelonia mydas* eggshells	Ecuador	^7^
	078 FUS		KC573938	KC573884	*Caretta caretta* embryo	Cape Verde	^7^
	079 FUS		KC573939	KC573885	*Caretta caretta* eggshells	Cape Verde	^7^
	099 FUS		KC573956	KC573886	*Caretta caretta* embryo	Cape Verde	^7^
*F. falciforme (cont.)*	142 FUS		KC573987	KC573887	*Chelonia mydas* eggshells	Ecuador	^7^
	181 FUS		KC573990	KC573888	*Natator depressus* eggshells	Australia	^7^
	182 FUS		KC573991	KC573889	*Natator depressus* eggshells	Australia	^7^
	209 FUS		KC574000	KC573890	*Lepidochelys olivacea* eggshells	Ecuador	^7^
	215 FUS		KC574002	KC573891	*Lepidochelys olivacea* eggshells	Ecuador	^7^
	219 FUS		KC574004	KC573892	*Lepidochelys olivacea* eggshells	Ecuador	^7^
	CBS 121450	JX435211	JX435211	JX435161	Declined grape vine	Syria	^28^
	CBS 124627	JX435184	JX435184	JX435134	Human nail	France	^28^
	CBS 475.67 T	MG189935	MG189915	LT906669	Human mycetoma	Puerto Rico	^28^
	ML16007	OM574603	ON237617	ON237631	*Caretta caretta* post-hatchling	South Africa	This study
	ML16008	OM574604	ON237618	ON237632	*Caretta caretta* post-hatchling	South Africa	This study
	ML16009	OM574605	ON237619	ON237633	*Caretta caretta* post-hatchling	South Africa	This study
	NRRL 22781	DQ094334	DQ236376	DQ246849	Human cornea	Venezuela	^28^
	NRRL 28562	DQ094376	DQ236418	DQ246903	Human bone	USA	^28^
	NRRL 28563	DQ094377	DQ236419	DQ246904	Clinical isolate	USA	^28^
	NRRL 28565		DQ094379	DQ236421	Human wound	USA	^1^
	NRRL 31162		DQ094392	DQ236434	Human	Texas	^1^
	NRRL 32307	DQ 094405	DQ236447	DQ246935	Human sputum	Unknown	^28^
	NRRL 32313	EU329678	EU329678	DQ246941	Human corneal ulcer	Unknown	^28^
	NRRL 32331	DQ094428	DQ236470	DQ246959	Human leg wound	Unknown	^28^
	NRRL 32339	DQ094436	DQ236478	DQ246967	Human	Unknown	^28^
	NRRL 32540	DQ094471	DQ236513	DQ247006	Human eye	India	^28^
	NRRL 32544	DQ094475	DQ23651	DQ247010	Human eye	India	^28^
	NRRL 32547	EU329680	EU329680	DQ247012	Human eye	India	^28^
	NRRL 32714	DQ094496	DQ236538	DQ247034	Human eye	USA	^28^
	NRRL 32718	DQ094500	DQ236542	DQ247038	Human eye	USA	^28^
	NRRL 32729	DQ094510	DQ236552	DQ247049	Human eye	USA	^28^
	NRRL 32738	DQ094519	DQ236561	DQ247058	Human eye	USA	^28^
	NRRL 32754	DQ094533	DQ236575	DQ247072	Turtle nare lesion	USA	^28^
	NRRL 32778	DQ094549	DQ236591	DQ247088	Equine corneal ulcer	USA	^28^
	NRRL 32798	DQ094567	DQ236609	DQ247107	Human	USA	^28^
	NRRL 43441	DQ790522	DQ790522	DQ790478	Human cornea	USA	^28^
	NRRL 43536	EF453118	EF453118	EF452966	Human cornea	USA	^28^
	NRRL 43537	DQ790550	DQ790550	DQ790506	Human cornea	USA	^28^
	NRRL 52832	GU170651	GU170651	GU170631	Human toenail	Italy	^28^
	NRRL 54966	KC808233	KC808233	KC808193	Equine eye	USA	^28^
	NRRL 54983	KC808248	KC808248	KC808206	Equine eye	USA	^28^
*F. gamsii*	CBS 143207 T	DQ094420	DQ236462	DQ246951	Human bronchoalveolar lavage fluid	USA	^28^
	NRRL 32794	DQ094563	DQ236605	DQ247103	Humidifier coolant	USA	^28^
	NRRL 43502	DQ790532	DQ790532	DQ790488	Human cornea	USA	^28^
*F. keratoplasticum*	001 AFUS		FR691753	JN939570	*Caretta caretta* embryo	Cape Verde	^7^
	001 CFUS		FR691754	KC594706	*Caretta caretta* embryo	Cape Verde	^7^
	009 FUS		FR691760	KC573903	*Caretta caretta* eggshells	Cape Verde	^7^
	010 FUS		FR691761	KC573904	*Caretta caretta* embryo	Cape Verde	^7^
	013 FUS		FR691764	KC573907	*Caretta caretta* eggshells	Cape Verde	^7^
	014 FUS		FR691757	KC573908	*Caretta caretta* eggshells	Cape Verde	^7^
	015 FUS		FR691759	KC573909	*Caretta caretta* eggshells	Cape Verde	^7^
*F. keratoplasticum (cont.)*	016 FUS		FR691758	KC573910	*Caretta caretta* eggshells	Cape Verde	^7^
	018 FUS		FR691765	KC573911	*Caretta caretta* eggshells	Cape Verde	^7^
	019 FUS		FR691766	KC573912	*Caretta caretta* eggshells	Cape Verde	^7^
	021 FUS		FR691768	KC573913	*Caretta caretta* embryo	Cape Verde	^7^
	028 FUS		KC573927	KC573914	*Chelonia mydas* eggshells	Ecuador	^7^
	029 FUS		KC573928	KC573915	*Eretmochelys imbricata* eggshells	Ecuador	^7^
	030 FUS		KC573929	KC573916	*Eretmochelys imbricata* eggshells	Ecuador	^7^
	034 FUS		KC573933	KC573918	*Eretmochelys imbricata* eggshells	Ecuador	^7^
	036 FUS		KC573935	KC573919	*Eretmochelys imbricata* eggshells	Ecuador	^7^
	223 FUS		KC574007	KC573920	*Eretmochelys imbricata* eggshells	Ascencion Island	^7^
	230 FUS		KC574010	KC573922	*Eretmochelys imbricata* eggshells	Ascencion Island	^7^
	CBS 490.63 T	LR583721	LR583929	LT906670	Human	Japan	^28^
	FMR 7989 = NRRL 46696	EU329705	EU329705	AM397219	Human eye	Brazil	^28^
	FMR 8482 = NRRL 46697	EU329706	EU329706	AM397224	Human tissue	Qatar	^28^
	FRC-S 2477 T	NR130690	JN235282	JN235712	Indoor plumbing	USA	^28^
	ML16001	OM574597	ON237611	ON237625	*Caretta caretta* post-hatchling	South Africa	This study
	ML16002	OM574598	ON237612	ON237626	*Caretta caretta* post-hatchling	South Africa	This study
	ML16003	OM574599	ON237613	ON237627	*Caretta caretta* post-hatchling	South Africa	This study
	ML16004	OM574600	ON237614	ON237628	*Caretta caretta* post-hatchling	South Africa	This study
	ML16005	OM574601	ON237615	ON237629	*Caretta caretta* post-hatchling	South Africa	This study
	ML16010	OM574606	ON237620	ON237634	*Caretta caretta* post-hatchling	South Africa	This study
	ML16013	OM574609	ON237623	ON237637	*Caretta caretta* post-hatchling	South Africa	This study
	ML16019	OM574610	ON237624	ON237638	*Caretta caretta post hatchling*	South Africa	This study
	NRRL 22640	DQ094327	DQ236369	DQ246842	Human cornea	Argentina	^28^
	NRRL 22791	DQ094337	DQ236379	DQ246853	Iguana tail	Unknown	^28^
	NRRL 28014	DQ094354	DQ236396	DQ246872	Human	USA	^28^
	NRRL 28561	DQ094375	DQ236417	DQ246902	Human wound	USA	^28^
	NRRL 32707	DQ094490	DQ236532	DQ247027	Human eye	USA	^28^
	NRRL 32710	DQ094492	DQ236534	DQ247030	Human eye	USA	^28^
	NRRL 32780	DQ094551	DQ236593	DQ247090	Sea turtle	USA	^28^
	NRRL 32838	EU329681	EU329681	DQ247144	Sea turtle	USA	^28^
	NRRL 32959	DQ094632	DQ236674	DQ247178	Manatee skin	USA	^28^
	NRRL 43443	EF453082	EF453082	EF453082	Human	Italy	^44^
	NRRL 43490	DQ790529	DQ790529	DQ790485	Human eye	USA	^28^
	NRRL 43649	EF453132	EF453132	EF452980	Human eye	USA	^28^
	NRRL 46437	GU170643	GU170643	GU170623	Human toenail	Italy	^28^
	NRRL 46438	GU170644	GU170644	GU170624	Human toenail	Italy	^28^
	NRRL 46443		GU170646	GU170646	Human foot	Italy	^45^
	NRRL 52704	JF740908	JF740908	JF740786	*Tetranychus urticae*	USA	^28^
*F. lichenicola*	CBS 279.34 T	LR583725	LR583933	LR583615	Human	Somalia	^28^
	CBS 483.96	LR583728	LR583936	LR583618	Air Brazil	Brazil	^28^
	CBS 623.92ET	LR583730	LR583938	LR583620	Human necrotic wound	Germany	^28^
	NRRL 28030	DQ094355	DQ236397	DQ246877	Human	Thailand	^28^
	NRRL 34123	DQ094645	DQ236687	DQ247192	Human eye	India	^28^
*F. metavorans*	CBS 135789 T	LR583738	LR583946	LR583627	Human pleural effusion	Greece	^28^
	NRRL 28018	LR583740	FJ240360	DQ246875	Human	USA	^28^
	NRRL 28019	LR583741	FJ240361	DQ246876	Human	USA	^28^
*F. parceramosum*	CBS 115695 T	JX435199	JX435199	JX435149	Soil	South Africa	^28^
	NRRL 31158	DQ094389	DQ236431	DQ246916	Human wound	USA	^28^
*F. petroliphilum*	NRRL 32304	DQ094402	DQ236444	DQ246932	Human nail	USA	^28^
	NRRL 32315	DQ094412	DQ236454	DQ246943	Human groin ulcer	USA	^28^
	NRRL 43812	EF453205	EF453205	EF453054	Contact lens solution	Unknown	^28^
*F. pseudensiforme*	CBS 241.93	JX435198	JX435198	JX435148	Human mycetoma	Suriname	^28^
	FRC-S 1834 = CBS 125729 T	KC691584	KC691584	DQ247512	Dead tree	Sri Lanka	^28^
*F. pseudotonkinense*	CBS 143038	LR583758	LR583962	LR583640	Human cornea	Netherlands	^28^
*F. quercicola*	NRRL 22611	DQ094326	DQ236368	DQ246841	Human cornea	USA	^28^
	NRRL 22652 T	LR583760	LR583964	DQ247634	*Quercus cerris*	Italy	^28^
	NRRL 32736	DQ094517	DQ236559	DQ247056	Human eye	USA	^28^
*N. solani*	CBS 112101	LR583772	LR583977	LR583653	Human vocal prosthesis	Belgium	^28^
	CBS 124893	JX435191	JX435191	JX435141	Human nail	France	^28^
	GJS 09-1466 T	KT313633	KT313633	KT313611	*Solanum tuberosum*	Slovenia	^28^
	NRRL 22779	DQ094333	DQ236375	DQ246848	Human toenail	New Zealand	^28^
	NRRL 31168	DQ094395	DQ236437	DQ246922	Human toe	USA	^28^
	NRRL 32492	EU329679	EU329679	DQ246990	Human	USA	^28^
	NRRL 32737	DQ094518	DQ236560	DQ247057	Human eye	USA	^28^
	NRRL 32791	DQ094560	DQ236602	DQ247100	Unknown	USA	^28^
	NRRL 32810	DQ094577	DQ236619	DQ247118	Human corneal ulcer	USA	^28^
	NRRL 43468	EF453093	EF453093	EF452941	Human eye	USA	^28^
	NRRL 43474	EF453097	EF453097	EF452945	Human eye	USA	^28^
	NRRL 44896	GU170639	GU170639	GU170619	Human toenail	Italy	^28^
	NRRL 46598	GU170648	GU170648	GU170628	Human toenail	Italy	^28^
*F. stericola*	CBS 142481 T	LR583779	LR583984	LR583658	Compost yard debris	Germany	^28^
	CBS 144388	LR583780	LR583985	LR583659	Greenhouse humic soil	Belgium	^28^
	CBS 260.54	LR583776	LR583981	LR583657	Unknown	Unknown	^28^
	NRRL 22239	LR583777	LR583982	DQ247562	Nematode egg	Germany	^28^
*F. suttonianum*	CBS 124892	JX435189	JX435189	JX435139	Human nail	Gabon	^28^
	CBS 143214 T	DQ094617	DQ236659	DQ247163	Human wound	USA	^28^
	NRRL 28000	DQ094348	DQ236390	DQ246866	Human	USA	^28^
	NRRL 32316	DQ094413	DQ236455	DQ246944	Human cornea	USA	^28^
	NRRL 54972	MG189940	MG189925	KC808197	Equine eye	USA	^28^
*F. tonkinense*	CBS 115.40 T	MG189941	MG189926	LT906672	*Musa sapientum*	Vietnam	^28^
	CBS 222.49	LR583783	LR583988	LR583661	*Euphorbia fulgens*	Netherlands	^28^
	NRRL 43811	EF453204	EF453204	EF453053	Human cornea	USA	^28^
*F. vasinfecta*	CBS 101957	LR583797	LR584002	LR583676	Human blood, sputum and wound	Germany	^28^
	CBS 446.93 T	LR583791	LR583996	LR583670	Soil	Japan	^28^
	NRRL 43467	EF453092	EF453092	EF452940	Human eye	USA	^28^
*Fusarium* sp. (AF1)	NRRL 22231	KC691570	KC691570	KC691542	Beetle on *Hevea brasiliensis*	Malaysia	^28^
	NRRL 46518	KC691571	KC691571	KC691543	Beetle on *Hevea brasiliensis*	Malaysia	^28^
	NRRL 46519	KC691572	KC69157	KC691544	Beetle on *Hevea brasiliensis*	Malaysia	^28^
*Fusarium* sp. (AF6)	NRRL 62590	KC691574	KC691574	KC691546	*Euwallacea fornicatus* on *Persea americana*	USA	^28^
	NRRL 62591	KC691573	KC691573	KC691545	*Euwallacea fornicatus* on *Persea americana*	USA	^28^
*Fusarium* sp. (AF7)	NRRL 62610	KC691575	KC691575	KC691547	*Euwallacea sp.* on *Persea ameri- cana*	Australia	^28^
	NRRL 62611	KC691576	KC691576	KC691548	*Euwallacea sp.* on *Persea ameri- cana*	Australia	^28^
*Fusarium* sp. (AF8)	NRRL 62585	KC691582	KC691582	KC691554	*Euwallacea fornicatus* on *Persea americana*	USA	^28^
	NRRL62584	KC691577	KC691577	KC691549	*Euwallacea fornicatus* on *Persea americana*	USA	^28^
*Fusarium* sp. (FSSC 12)	NRRL 22642	DQ094329	DQ236371	DQ246844	*Penaceous japonicus* gill	Japan	^28^
	NRRL 25392	EU329672	EU329672	DQ246861	American lobster	USA	^28^
	NRRL 32309	DQ094407	DQ236449	DQ246937	Sea turtle	USA	^28^
	NRRL 32317	DQ094414	DQ236456	DQ246945	Treefish	USA	^28^
	NRRL 32821	DQ094587	DQ236629	DQ247128	Turtle egg	USA	^28^

### Morphological observation

Agar plugs (6 mm diameter) of the selected isolates were transferred onto fresh PDA and Carnation leaf agar (CLA) plates and incubated at 26 °C ± 1 °C for 7 and 21 days, respectively for further morphological characterisation. Morphological characterisation was based on the taxonomic keys of Leslie and Summerell, 2006^35^. Gross macro-morphology of all isolates was examined on PDA after 7 days, this comprised (i) colony colour on top of the plate, (ii) colony colour on the reverse side (iii) colony size and (iv) texture of the hyphal growth. With a primary focus on 3 strains namely ML16006, ML16011 and ML16012.

Micro-morphological evaluation of the respective isolates was achieved by examining CLA plates in situ under the 20X or 40X objective, using a Nikon eclipse N*i* compound microscope. The following characteristics were noted: (i) microconidia; shape, size, number of septa and their arrangement on phialide cells (ii) macroconidia; shape, size, number of septa and the shape of the apical and basal cells (iii) sporodachia; when present colour was noted and (iv) chlamydospores; texture of cell walls, position on hyphae and the arrangements. The length and the width of 30 micro- and macroconidia were measured for each isolate (Online Resource 1). The oval shape of the microconidia was measured by drawing a straight line from top to the bottom for the length and the width was measured across the septa or when no septa was observed, at the widest part of the cell. The length of the macroconidia was measured by drawing a straight line from the apical side of the cell to the basal side of the cell. The width was measured at the apical side of the middle septa. Conidia and chlamydospores were mounted on glass slides using water as mounting medium from fungal structures grown on carnation leaf agar^36^ and photographed.

All methods were carried out in accordance with relevant guidelines and regulations. All experimental protocols were approved by a named institutional and/or licensing committee.

## Results

### Molecular characterisation and Phylogenetic analyses

**Figure 2:**
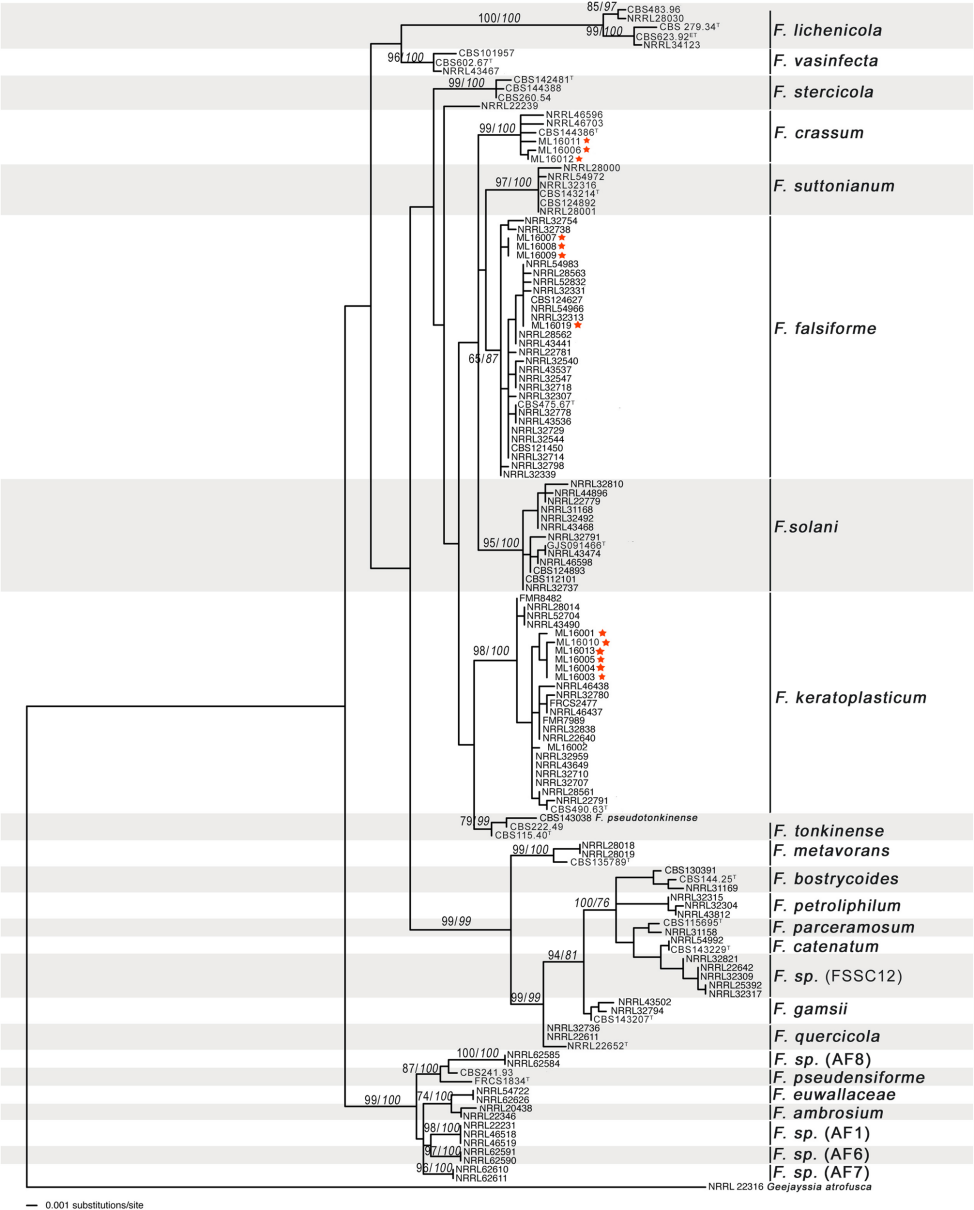
Maximum likelihood analysis of *Fusarium* species isolates based on three loci, translation elongation factor 1 α (*tef1*), large subunit (LSU) and internal transcribed standard (ITS). Numbers within the tree represent the bootstrap values of 1 000 replicates, followed by the posterior probability (italics). Strains isolated during this study are marked with a red asterisk (*).

**Figure 3:**
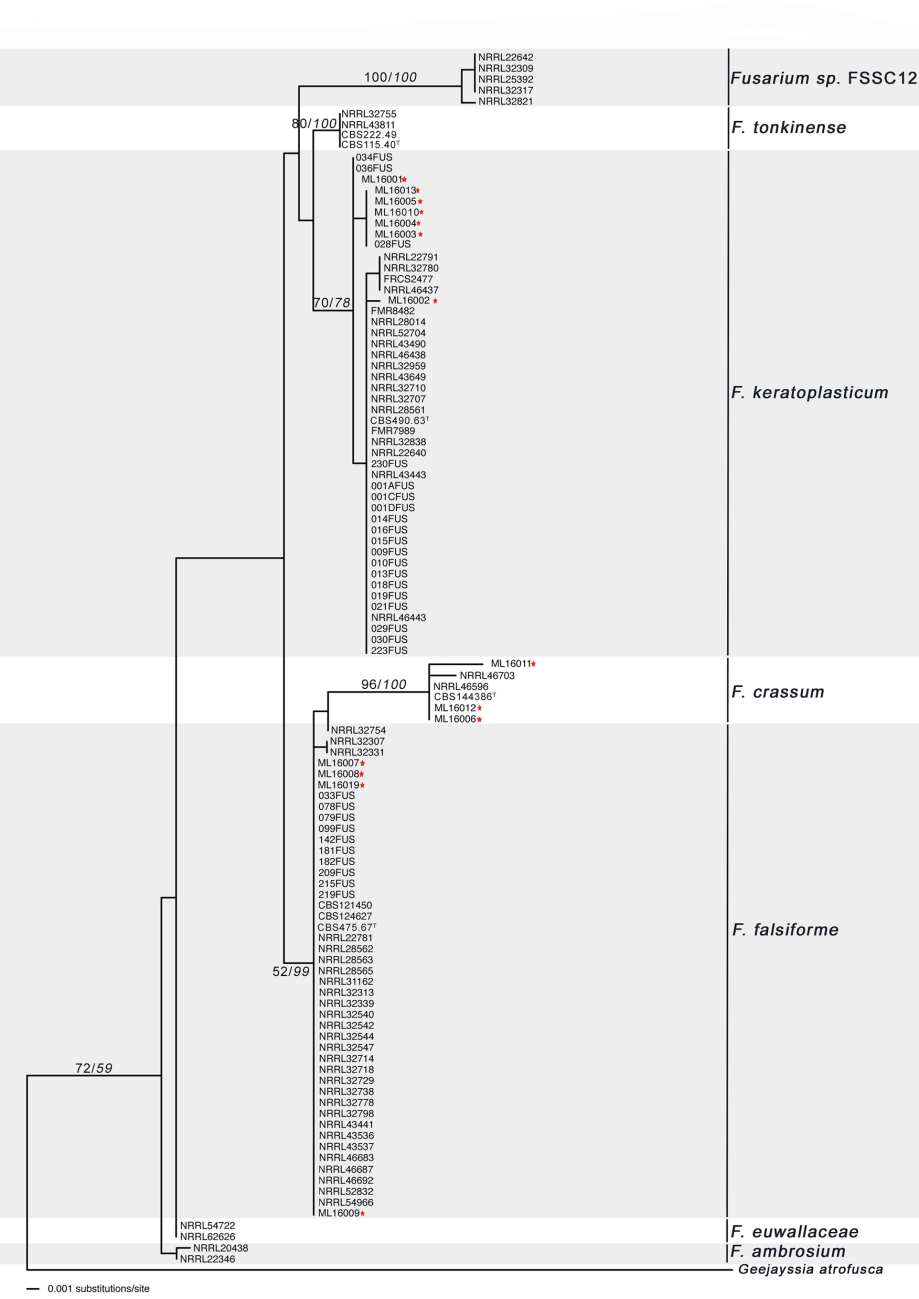
Maximum likelihood analysis of *Fusarium* species isolates from other marine animals based on two loci, large subunit (LSU) and internal transcribed standard (ITS). Numbers within the tree represent the bootstrap values of 1 000 replicates, followed by the posterior probability (italics). Strains isolated during this study are marked with a red asterisk (*).

Phylogenetic analyses (Figs. 2 and 3) showed 3 (*F. falciforme, F. keratoplasticum* and *F. crassum*) distinct species. A phylogenetic tree generated from the combined dataset of LSU, ITS and *tef1* gene regions, represented 3 lineages within the *Fusarium solani* species complex (FSSC). The maximum likelihood (ML) analysis included 135 taxa (including the outgroup). In the analyses, 14 strains isolated during this study, aligned with three species within *Fusarium.* Seven strains (ML16001; ML16013; ML16005; ML16004; ML16003; ML16002; ML16010) grouped with the *F. keratoplasticum* clade with a strong bootstrap support. Four strains (ML16007; ML16008; ML16009; ML16019) grouped within the more diverse *F. falciforme* clade. Another three strains (ML16006; ML16011; ML16012) grouped with *F. crassum.* Secondary phylogenetic analysis of the ITS and LSU gene regions, included 118 taxa (including the outgroup). These analyses confirmed the findings of primary phylogenetic analyses and showed that isolates from this study aligned with isolates that were previously associated with turtles and turtle eggs.

### Morphological observation

**Figure 4:**
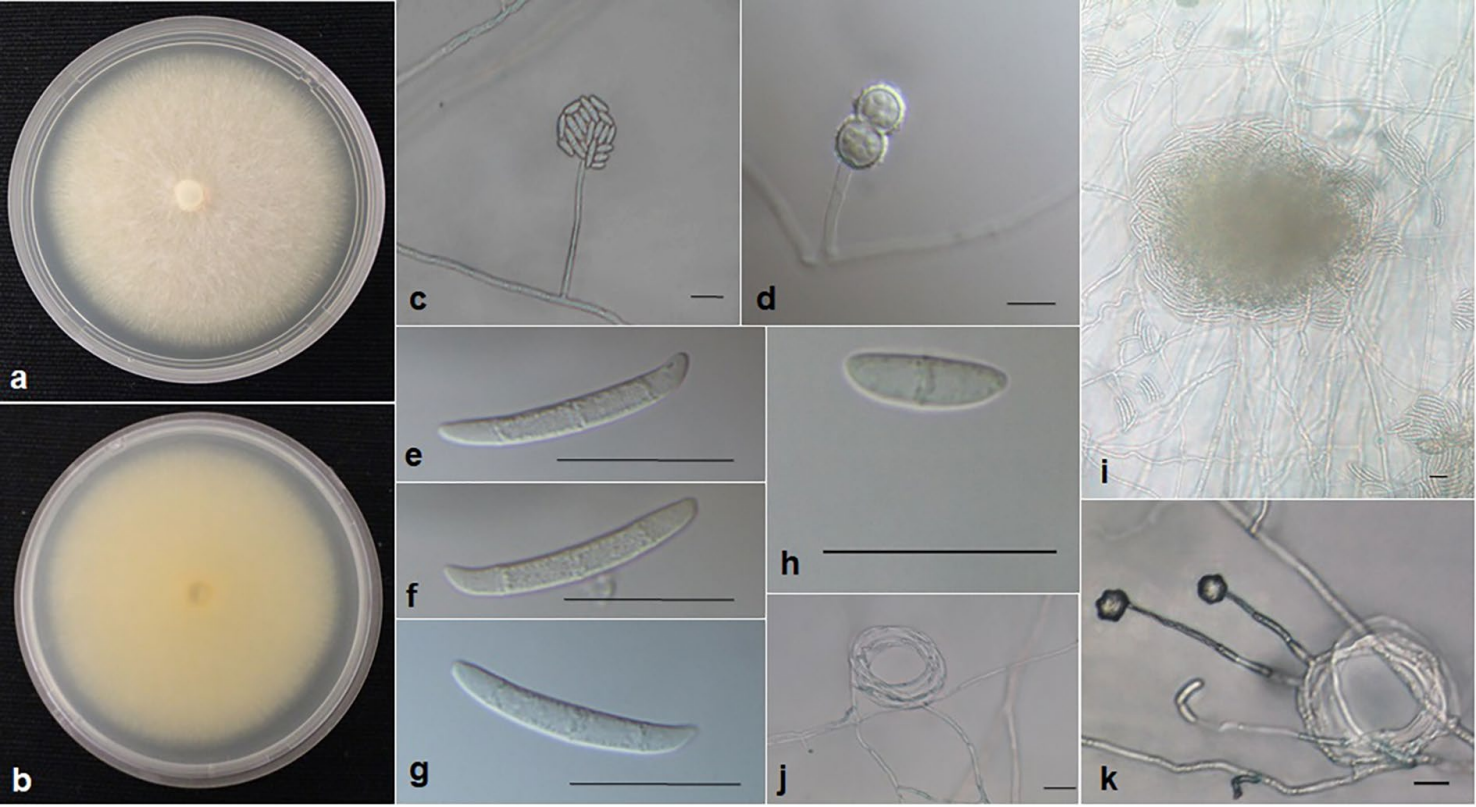
*Fusarium crassum*, (**a**) Colony on PDA and (**b**) reverse side after 7 days incubation at 26 ± 1 °C. (**c**) areal mycelia presenting microconidia in false heads in situ, (**d**) Chlamydospores with rough walls in situ*,* (**e**–**g**) macroconidia, (**h**) microconidia with one septa, (**i**) macroconidia in situ on carnation leaf agar after 21 days incubation at 26 ± 1 °C, (**j**–**k**) hyphal coils observed in situ on carnation leaf agar. All scale bars = 20 µm.

Three strains expressed significant different morphological characteristics compared to other strains isolated during this study. These three strains were relatively fast growing on PDA, reaching a colony size of 70–75 mm diameter after 7 days of incubation at 26 ± 1 °C. White, flat floccose mycelium with light peach to yellow centre. White to pale light yellow on the reverse side. On CLA, incubated at 26 ± 1 °C, reaching a colony size of 80–90 mm diameter in 7 days. Microconidia were oval, ellipsoidal to sub-cylindrical in shape, with 0–1 septum, smooth and thin walled arranged in false heads at the tip of long monophialides. Average aseptate microconidia measured as follows for the three strains (n = 30 per strain); 11.5 µm (± 1.25) × 4.00 µm (± 0.5), 12.0 µm (± 1.0) × 4.0 µm (± 0.5) and 11.5 µm (± 2.0) × 4.25 µm (± 0.4). Microconidia with one septa measured as follows (n = 30 per strain); 15.0 µm (± 2.0) × 4.25 µm (± 0.5), 15.0 µm (± 1.5) × 4.0 µm (± 0.5) and 15.5 µm (± 5.0) × 4.5 µm (± 0.5). Macroconidia were fusiform in shape with the dorsal sides more curved than the ventral sides, blunt apical cells and barely notched foot cells. Macroconidia consisted of 3–4 septa and measured as follows (n = 30 per strain); 31.5 µm (± 3.0) × 5.0 µm (± 0.5), 32.0 µm (± 2.0) × 5.0 µm (± 0.5) and 30.0 µm (± 1.0) × 5.0 µm (± 0.5). Sporodochia ranged from clear to beige in colour. Chlamydospores were first observed after 14 days of incubation on CLA plates, and were globose in shape with rough walls, positioned terminally, sometimes single but mostly in pairs. Distinct hyphal coils were observed in all three strains (Fig. 4). The morphology is consistent with that described for *N. crassum*^28^ (Fig. 4).

## Discussion

*Fusarium* infections, specifically *F. keratoplasticum* and *F. falciforme* have been reported from infected eggs and embryos of turtle species, including endangered species, at major nesting sites along the Atlantic, Pacific and Indian Oceans, as well as the Mediterranean and Caribbean Sea^15,16,18,20,21,37^. Management strategies to mitigate emerging fungal diseases, like *Fusarium* infections in turtle eggs, are influenced by identifying whether a pathogen is novel or endemic and the understanding of its ecology and distribution. A novel pathogen gains access to and infects naïve hosts as a result of migration of the pathogen or the development of novel pathogenic genotypes, in contrast endemic pathogens occur naturally in the host’s environment, but shifts in environmental conditions and/or host susceptibility influence pathogenicity^37^. Thus, effective management strategies to mitigate novel pathogens should aim at preventing pathogen introduction and expansion, while disease caused by endemic pathogens relies on an understanding of environmental and host factors that influence disease emergence and severity^37^. Phylogenetic analysis provides important information to assist in understanding the ecology, introduction and distribution of infectious agents^37,38^. The first aim of this study was to use multigene phylogenetic analyses to identify *Fusarium* strains isolated from the carapace, flippers, head, and neck area of post-hatchling loggerhead sea turtles (*Caretta caretta*) with fungal skin infections that stranded along the South African coastline and kept at a rehabilitation centre. The genus *Fusarium* was recently revised, with an attempt to standardise the taxonomy and nomenclature after a lack of formal species descriptions, Latin names and nomenclatural type specimens were identified^39^. Strains from this study grouped with three *Fusarium* species of which two species, *F. keratoplasticum* and *F. falciforme,* were previously reported to occur on animal hosts, including turtles. The third species, *F. crassum* is rather surprising as this species is only known from a human toenail and nematode eggs, while the origin of the type strain is unknown. Three strains (ML16011, ML16012 and ML16006) grouped with two *F. crassum* strains*.* Strain identifications were confirmed with the morphological characteristics that agreed with species descriptions published in 2019^28^, with the one exception of chlamydospore wall texture for *F. crassum*. Chlamydospore walls in this study for all three *F. crassum* strains were smooth, while previously it has been documented with a rough texture.

Turtle egg fusariosis (STEF) is a disease that has increasingly been reported over the last decade and is considered a potential conservation threat to six out of seven species of marine turtles^16,37^. Skin disease and systemic infections caused by *Fusarium* species has been reported in adult and subadult turtles and in captive reared hatchlings^5,11–15,19^, but has not been reported in post-hatchling loggerhead sea turtles (*C. caretta*) undergoing rehabilitation. Clinical signs reported in juvenile, subadult and adult loggerhead sea turtles (*C. caretta*) with *Fusarium* infections were localised and generalised lesions of the skin and carapace, consisting of areas of discolouration and loss of shell^12^. Clinical signs observed in post-hatchling loggerhead sea turtles (*C. caretta*) in this study were similar, but generalised sloughing of scales on the limbs and head, and a soft, crumbly carapace and plastron were more common than focal lesions. Histopathology was not performed in this study to confirm the association of fungal hyphae with pathological changes in the skin, and, therefore, the role of the *Fusarium* isolates in the skin lesions cannot definitively be identified (as isolation of fungus could be from normal skin flora or the environment), however, fungal hyphae, often in dense mats, were seen in epidermal scrapes from affected turtles (Online Resource 2). Although *Fusarium* isolates (and other fungi) have been identified in the skin of healthy adult *C. caretta*^12^, a finding of numerous hyphae (hyphal mats) in skin scrapings would not be considered a normal finding in healthy turtle skin and thus it is considered likely that the fungal elements observed, and therefore the isolates identified, were associated with the observed pathology. The epidemiology of turtle pathogenic isolates *F. keratoplasticum* and *F. falciforme* in sea turtle nesting sites are not fully understood^37^, however, it has been suggested that tank substrates and/or biofilms forming in the water supply infrastructure or filtering systems may act as a source of infection, to traumatised and immunocompromised sea turtles^11,12,40^.

Investigations into the source of infection were not undertaken in this study, so it is not clear if the fungal isolates originated in the rehabilitation environment or were present in the skin on admission. Cafarchia and colleagues (2019) found increased length of stay to be a risk factor for fungal colonisation, where turtles staying in a rehabilitation centre for over 20 days were more frequently colonised with *Fusarium*^12^. Loggerhead sea turtles (*C. caretta*) in this study exhibited clinical signs around 20–30 days after admission and it is likely that most individuals experienced some degree of immunocompromise in the initial stages of rehabilitation. This, combined with physical skin trauma that may be present on admission may have provided a suitable environment for fungal colonisation. The second aim of study was to establish the phylogenetic relationship between *F. keratoplasticum* and *F. falciforme* strains isolated during this study and strains that were previously associated with brood failure and high mortality rates^17,18^. Combined sequence data of the ITS and LSU regions revealed that seven of the strains formed part of the monophyletic *F. keratoplasticum* clade. Strains isolated during this study showed a close phylogenetic relation with other species in this clade, consisting of species that were previously isolated from Hawksbill (*E. imbricata*) and green sea turtle (*C. mydas*) eggs shells from nesting beaches along the Pacific Ocean in Ecuador^7,16^. Furthermore, phylogenetic analyses of the *F. falciforme* group showed close resemblance to strains that were previously isolated from olive ridley sea turtle (*L. olivacea*), green sea turtle (*C. mydas*), flatback sea turtle (*N. depressus*) and loggerhead sea turtle (*C. caretta*) egg shells and *C. caretta* embryos on nesting beaches in Australia, Cape Verde and Ecuador, Turkey, along the Pacific, Atlantic and Indian Ocean^7,15–17,20,21^. In addition, these strains showed a close resemblance to a strain that was previously isolated from a lesion in an adult turtle nare from the USA^29^. Based on the ITS and LSU gene regions, a genetic relationship exists between *Fusarium* species associated with turtle egg infections (also known as STEF) and *Fusarium* species isolated from post-hatchling loggerhead sea turtles (*C. caretta*) that stranded on beaches in South Africa along the Indian ocean.

Infections caused by members of this genus have been reported in numerous other aquatic animals in the past^6,9,10^, but for many of these, identification has been limited and mostly based on morphological characteristics. Many reports based on morphology only identified causative agents as *Fusarium* (*F. solani*)*,* lacking further identification. Accurate identification of pathogenic *Fusarium* members is essential for epidemiological purposes and for assisting in management programs, however, more research is required to complete the puzzle and fully understand the ecology and distribution of these pathogens, especially amongst reptiles and aquatic animals. This is the first confirmed record of *F. keratoplasticum* and *F. falciforme* strains isolated from post-hatchling loggerhead sea turtles (*Caretta caretta*) from the South African coastline that were not associated with nesting sites. This is also the first record of *F. crassum* to be associated with loggerhead sea turtles.

## Supplementary Information

Supplementary Legends.

Supplementary Figure 1.

Supplementary Figure 2.
