# Publisher Correction: *Fusarium* species isolated from post-hatchling loggerhead sea turtles (*Caretta caretta*) in South Africa

**DOI:** 10.1038/s41598-022-12820-2

**Published:** 2022-05-27

**Authors:** Mariska R. Greeff-Laubscher, Karin Jacobs

**Affiliations:** 1 Water Research Group, Unit Environmental Sciences and Management, Potchefstroom Campus, North-West University, Private Bag X6001, Potchefstroom, 2520 South Africa; 2 Department of Microbiology, Stellenbosch University, Stellenbosch, 7599 South Africa

Correction to: *Scientific Reports* 10.1038/s41598-022-06840-1, Published online 07 April 2022

The original version of this Article contained errors in Table 2, where the LSU and EF GenBank accession numbers for the species ‘*F. crissum’, ‘F. falciforme’* and ‘*F. keratoplasticum’* were omitted.

**Table 2:** *Fusarium* strains included in the phylogenetic analyses.

Species name	Strain number	Genbank accession number			Source	Origin	Reference
		ITS	LSU	EF			
*Geejayessia atrofusca (outgroup)*	NRRL 22316	AF178423	AF178392	AF178361	*Staphylea trifolia*	USA	^28^
*F. ambrosium*	NRRL 20438	AF178397	DQ236357	AF178332	*Euwallacea fornicatus* on *Camellia sinensis*	India	^28^
	NRRL 22346 = CBS 571.94ET	EU329669	EU329669	FJ240350	*Euwallacea fornicatus* on *Camellia sinensis*	India	^28^
*F. bostrycoides*	CBS 130391	EU329716	EU329716	HM347127	Human eye	Brazil	^28^
	CBS144.25NT	LR583704	LR583912	LR583597	Soil	Honduras	^28^
	NRRL 31169	DQ094396	DQ236438	DQ246923	Human oral wound	USA	^28^
*F. catenatum*	CBS 143229 T = NRRL54993	KC808256	KC808256	KC808214	*Stegostoma fasciatum* multiple tissues	USA	^28^
	NRRL 54992	KC808255	KC808255	KC808213	*Stegostoma fasciatum* multiple tissues	USA	^28^
*F. crassum*	CBS 144386 T	LR583709	LR583917	LR583604	Unknown	France	^28^
	NRRL 46596	GU170647	GU170647	GU170627	Human toenail	Italy	^28^
	NRRL 46703	EU329712	EU329712	HM347126	Nematode egg	Spain	^28^
	ML16006	OM574602			*Caretta caretta* post-hatchling	South Africa	This study
	ML16011	OM574607			*Caretta caretta* post-hatchling	South Africa	This study
	ML16012	OM574608			*Caretta caretta* post-hatchling	South Africa	This study
*F. euwallaceae*	NRRL 54722 = CBS 135854 T	JQ038014	JQ038014	JQ038007	*Euwallacea fornicatus* on *Persea americana*	Israel	^28^
	NRRL 62626	KC691560	KC691560	KC691532	*Euwallacea fornicatus* on *Persea americana*	USA	^28^
*F. falciforme*	033 FUS		KC573932	KC573883	*Chelonia mydas* eggshells	Ecuador	^7^
	078 FUS		KC573938	KC573884	*Caretta caretta* embryo	Cape Verde	^7^
	079 FUS		KC573939	KC573885	*Caretta caretta* eggshells	Cape Verde	^7^
	099 FUS		KC573956	KC573886	*Caretta caretta* embryo	Cape Verde	^7^
*F. falciforme* (cont.)	142 FUS		KC573987	KC573887	*Chelonia mydas* eggshells	Ecuador	^7^
	181 FUS		KC573990	KC573888	*Natator depressus* eggshells	Australia	^7^
	182 FUS		KC573991	KC573889	*Natator depressus* eggshells	Australia	^7^
	209 FUS		KC574000	KC573890	*Lepidochelys olivacea* eggshells	Ecuador	^7^
	215 FUS		KC574002	KC573891	*Lepidochelys olivacea* eggshells	Ecuador	^7^
	219 FUS		KC574004	KC573892	*Lepidochelys olivacea* eggshells	Ecuador	^7^
	CBS 121450	JX435211	JX435211	JX435161	Declined grape vine	Syria	^28^
	CBS 124627	JX435184	JX435184	JX435134	Human nail	France	^28^
	CBS 475.67 T	MG189935	MG189915	LT906669	Human mycetoma	Puerto Rico	^28^
	ML16007	OM574603			*Caretta caretta* post-hatchling	South Africa	This study
	ML16008	OM574604			*Caretta caretta* post-hatchling	South Africa	This study
	ML16009	OM574605			*Caretta caretta* post-hatchling	South Africa	This study
	NRRL 22781	DQ094334	DQ236376	DQ246849	Human cornea	Venezuela	^28^
	NRRL 28562	DQ094376	DQ236418	DQ246903	Human bone	USA	^28^
	NRRL 28563	DQ094377	DQ236419	DQ246904	Clinical isolate	USA	^28^
	NRRL 28565		DQ094379	DQ236421	Human wound	USA	^1^
	NRRL 31162		DQ094392	DQ236434	Human	Texas	^1^
	NRRL 32307	DQ 094405	DQ236447	DQ246935	Human sputum	Unknown	^28^
	NRRL 32313	EU329678	EU329678	DQ246941	Human corneal ulcer	Unknown	^28^
	NRRL 32331	DQ094428	DQ236470	DQ246959	Human leg wound	Unknown	^28^
	NRRL 32339	DQ094436	DQ236478	DQ246967	Human	Unknown	^28^
	NRRL 32540	DQ094471	DQ236513	DQ247006	Human eye	India	^28^
	NRRL 32544	DQ094475	DQ23651	DQ247010	Human eye	India	^28^
	NRRL 32547	EU329680	EU329680	DQ247012	Human eye	India	^28^
	NRRL 32714	DQ094496	DQ236538	DQ247034	Human eye	USA	^28^
*F. falciforme* (cont.)	NRRL 32718	DQ094500	DQ236542	DQ247038	Human eye	USA	^28^
	NRRL 32729	DQ094510	DQ236552	DQ247049	Human eye	USA	^28^
	NRRL 32738	DQ094519	DQ236561	DQ247058	Human eye	USA	^28^
	NRRL 32754	DQ094533	DQ236575	DQ247072	Turtle nare lesion	USA	^28^
	NRRL 32778	DQ094549	DQ236591	DQ247088	Equine corneal ulcer	USA	^28^
	NRRL 32798	DQ094567	DQ236609	DQ247107	Human	USA	^28^
	NRRL 43441	DQ790522	DQ790522	DQ790478	Human cornea	USA	^28^
	NRRL 43536	EF453118	EF453118	EF452966	Human cornea	USA	^28^
	NRRL 43537	DQ790550	DQ790550	DQ790506	Human cornea	USA	^28^
	NRRL 52832	GU170651	GU170651	GU170631	Human toenail	Italy	^28^
	NRRL 54966	KC808233	KC808233	KC808193	Equine eye	USA	^28^
	NRRL 54983	KC808248	KC808248	KC808206	Equine eye	USA	^28^
*F. gamsii*	CBS 143207 T	DQ094420	DQ236462	DQ246951	Human bronchoalveolar lavage fluid	USA	^28^
	NRRL 32794	DQ094563	DQ236605	DQ247103	Humidifier coolant	USA	^28^
	NRRL 43502	DQ790532	DQ790532	DQ790488	Human cornea	USA	^28^
*F. keratoplasticum*	001 AFUS		FR691753	JN939570	*Caretta caretta* embryo	Cape Verde	^7^
	001 CFUS		FR691754	KC594706	*Caretta caretta* embryo	Cape Verde	^7^
	009 FUS		FR691760	KC573903	*Caretta caretta* eggshells	Cape Verde	^7^
	010 FUS		FR691761	KC573904	*Caretta caretta* embryo	Cape Verde	^7^
	013 FUS		FR691764	KC573907	*Caretta caretta* eggshells	Cape Verde	^7^
	014 FUS		FR691757	KC573908	*Caretta caretta* eggshells	Cape Verde	^7^
	015 FUS		FR691759	KC573909	*Caretta caretta* eggshells	Cape Verde	^7^
*F. keratoplasticum* (cont)	016 FUS		FR691758	KC573910	*Caretta caretta* eggshells	Cape Verde	^7^
	018 FUS		FR691765	KC573911	*Caretta caretta* eggshells	Cape Verde	^7^
	019 FUS		FR691766	KC573912	*Caretta caretta* eggshells	Cape Verde	^7^
	021 FUS		FR691768	KC573913	*Caretta caretta* embryo	Cape Verde	^7^
	028 FUS		KC573927	KC573914	*Chelonia mydas* eggshells	Ecuador	^7^
	029 FUS		KC573928	KC573915	*Eretmochelys imbricata* eggshells	Ecuador	^7^
	030 FUS		KC573929	KC573916	*Eretmochelys imbricata* eggshells	Ecuador	^7^
	034 FUS		KC573933	KC573918	*Eretmochelys imbricata* eggshells	Ecuador	^7^
	036 FUS		KC573935	KC573919	*Eretmochelys imbricata* eggshells	Ecuador	^7^
	223 FUS		KC574007	KC573920	*Eretmochelys imbricata* eggshells	Ascencion Island	^7^
	230 FUS		KC574010	KC573922	*Eretmochelys imbricata* eggshells	Ascencion Island	^7^
	CBS 490.63 T	LR583721	LR583929	LT906670	Human	Japan	^28^
	FMR 7989 = NRRL 46696	EU329705	EU329705	AM397219	Human eye	Brazil	^28^
	FMR 8482 = NRRL 46697	EU329706	EU329706	AM397224	Human tissue	Qatar	^28^
	FRC-S 2477 T	NR130690	JN235282	JN235712	Indoor plumbing	USA	^28^
	ML16001	OM574597			*Caretta caretta* post-hatchling	South Africa	This study
	ML16002	OM574598			*Caretta caretta* post-hatchling	South Africa	This study
	ML16003	OM574599			*Caretta caretta* post-hatchling	South Africa	This study
	ML16004	OM574600			*Caretta caretta* post-hatchling	South Africa	This study
	ML16005	OM574601			*Caretta caretta* post-hatchling	South Africa	This study
	ML16010	OM574606			*Caretta caretta* post-hatchling	South Africa	This study
	ML16013	OM574609			*Caretta caretta* post-hatchling	South Africa	This study
	ML16019	OM574610			*Caretta caretta post hatchling*	South Africa	This study
*F. keratoplasticum* (cont)	NRRL 22640	DQ094327	DQ236369	DQ246842	Human cornea	Argentina	^28^
	NRRL 22791	DQ094337	DQ236379	DQ246853	Iguana tail	Unknown	^28^
	NRRL 28014	DQ094354	DQ236396	DQ246872	Human	USA	^28^
	NRRL 28561	DQ094375	DQ236417	DQ246902	Human wound	USA	^28^
	NRRL 32707	DQ094490	DQ236532	DQ247027	Human eye	USA	^28^
	NRRL 32710	DQ094492	DQ236534	DQ247030	Human eye	USA	^28^
	NRRL 32780	DQ094551	DQ236593	DQ247090	Sea turtle	USA	^28^
	NRRL 32838	EU329681	EU329681	DQ247144	Sea turtle	USA	^28^
	NRRL 32959	DQ094632	DQ236674	DQ247178	Manatee skin	USA	^28^
	NRRL 43443	EF453082	EF453082	EF453082	Human	Italy	^44^
	NRRL 43490	DQ790529	DQ790529	DQ790485	Human eye	USA	^28^
	NRRL 43649	EF453132	EF453132	EF452980	Human eye	USA	^28^
	NRRL 46437	GU170643	GU170643	GU170623	Human toenail	Italy	^28^
	NRRL 46438	GU170644	GU170644	GU170624	Human toenail	Italy	^28^
	NRRL 46443		GU170646	GU170646	Human foot	Italy	^45^
	NRRL 52704	JF740908	JF740908	JF740786	*Tetranychus urticae*	USA	^28^
*F. lichenicola*	CBS 279.34 T	LR583725	LR583933	LR583615	Human	Somalia	^28^
	CBS 483.96	LR583728	LR583936	LR583618	Air Brazil	Brazil	^28^
	CBS 623.92ET	LR583730	LR583938	LR583620	Human necrotic wound	Germany	^28^
	NRRL 28030	DQ094355	DQ236397	DQ246877	Human	Thailand	^28^
	NRRL 34123	DQ094645	DQ236687	DQ247192	Human eye	India	^28^
*F. metavorans*	CBS 135789 T	LR583738	LR583946	LR583627	Human pleural effusion	Greece	^28^
	NRRL 28018	LR583740	FJ240360	DQ246875	Human	USA	^28^
	NRRL 28019	LR583741	FJ240361	DQ246876	Human	USA	^28^
*F. parceramosum*	CBS 115695 T	JX435199	JX435199	JX435149	Soil	South Africa	^28^
	NRRL 31158	DQ094389	DQ236431	DQ246916	Human wound	USA	^28^
*F. petroliphilum*	NRRL 32304	DQ094402	DQ236444	DQ246932	Human nail	USA	^28^
	NRRL 32315	DQ094412	DQ236454	DQ246943	Human groin ulcer	USA	^28^
	NRRL 43812	EF453205	EF453205	EF453054	Contact lens solution	Unknown	^28^
*F. pseudensiforme*	CBS 241.93	JX435198	JX435198	JX435148	Human mycetoma	Suriname	^28^
	FRC-S 1834 = CBS 125729 T	KC691584	KC691584	DQ247512	Dead tree	Sri Lanka	^28^
*F. pseudotonkinense*	CBS 143038	LR583758	LR583962	LR583640	Human cornea	Netherlands	^28^
*F. quercicola*	NRRL 22611	DQ094326	DQ236368	DQ246841	Human cornea	USA	^28^
	NRRL 22652 T	LR583760	LR583964	DQ247634	*Quercus cerris*	Italy	^28^
	NRRL 32736	DQ094517	DQ236559	DQ247056	Human eye	USA	^28^
*N. solani*	CBS 112101	LR583772	LR583977	LR583653	Human vocal prosthesis	Belgium	^28^
	CBS 124893	JX435191	JX435191	JX435141	Human nail	France	^28^
	GJS 09–1466 T	KT313633	KT313633	KT313611	*Solanum tuberosum*	Slovenia	^28^
	NRRL 22779	DQ094333	DQ236375	DQ246848	Human toenail	New Zealand	^28^
	NRRL 31168	DQ094395	DQ236437	DQ246922	Human toe	USA	^28^
	NRRL 32492	EU329679	EU329679	DQ246990	Human	USA	^28^
	NRRL 32737	DQ094518	DQ236560	DQ247057	Human eye	USA	^28^
	NRRL 32791	DQ094560	DQ236602	DQ247100	Unknown	USA	^28^
	NRRL 32810	DQ094577	DQ236619	DQ247118	Human corneal ulcer	USA	^28^
	NRRL 43468	EF453093	EF453093	EF452941	Human eye	USA	^28^
	NRRL 43474	EF453097	EF453097	EF452945	Human eye	USA	^28^
	NRRL 44896	GU170639	GU170639	GU170619	Human toenail	Italy	^28^
	NRRL 46598	GU170648	GU170648	GU170628	Human toenail	Italy	^28^
*F. stericola*	CBS 142481 T	LR583779	LR583984	LR583658	Compost yard debris	Germany	^28^
	CBS 144388	LR583780	LR583985	LR583659	Greenhouse humic soil	Belgium	^28^
	CBS 260.54	LR583776	LR583981	LR583657	Unknown	Unknown	^28^
	NRRL 22239	LR583777	LR583982	DQ247562	Nematode egg	Germany	^28^
*F. suttonianum*	CBS 124892	JX435189	JX435189	JX435139	Human nail	Gabon	^28^
	CBS 143214 T	DQ094617	DQ236659	DQ247163	Human wound	USA	^28^
	NRRL 28000	DQ094348	DQ236390	DQ246866	Human	USA	^28^
	NRRL 32316	DQ094413	DQ236455	DQ246944	Human cornea	USA	^28^
	NRRL 54972	MG189940	MG189925	KC808197	Equine eye	USA	^28^
*F. tonkinense*	CBS 115.40 T	MG189941	MG189926	LT906672	*Musa sapientum*	Vietnam	^28^
	CBS 222.49	LR583783	LR583988	LR583661	*Euphorbia fulgens*	Netherlands	^28^
	NRRL 43811	EF453204	EF453204	EF453053	Human cornea	USA	^28^
*F. vasinfecta*	CBS 101957	LR583797	LR584002	LR583676	Human blood, sputum and wound	Germany	^28^
	CBS 446.93 T	LR583791	LR583996	LR583670	Soil	Japan	^28^
	NRRL 43467	EF453092	EF453092	EF452940	Human eye	USA	^28^
*Fusarium* sp. (AF1)	NRRL 22231	KC691570	KC691570	KC691542	Beetle on *Hevea brasiliensis*	Malaysia	^28^
	NRRL 46518	KC691571	KC691571	KC691543	Beetle on *Hevea brasiliensis*	Malaysia	^28^
	NRRL 46519	KC691572	KC69157	KC691544	Beetle on *Hevea brasiliensis*	Malaysia	^28^
*Fusarium* sp. (AF6)	NRRL 62590	KC691574	KC691574	KC691546	*Euwallacea fornicatus* on *Persea americana*	USA	^28^
	NRRL 62591	KC691573	KC691573	KC691545	*Euwallacea fornicatus* on *Persea americana*	USA	^28^
*Fusarium* sp. (AF7)	NRRL 62610	KC691575	KC691575	KC691547	*Euwallacea sp.* on *Persea ameri- cana*	Australia	^28^
	NRRL 62611	KC691576	KC691576	KC691548	*Euwallacea sp.* on *Persea ameri- cana*	Australia	^28^
*Fusarium* sp. (AF8)	NRRL 62585	KC691582	KC691582	KC691554	*Euwallacea fornicatus* on *Persea americana*	USA	^28^
	NRRL62584	KC691577	KC691577	KC691549	*Euwallacea fornicatus* on *Persea americana*	USA	^28^
*Fusarium* sp. (FSSC 12)	NRRL 22642	DQ094329	DQ236371	DQ246844	*Penaceous japonicus* gill	Japan	^28^
	NRRL 25392	EU329672	EU329672	DQ246861	American lobster	USA	^28^
	NRRL 32309	DQ094407	DQ236449	DQ246937	Sea turtle	USA	^28^
	NRRL 32317	DQ094414	DQ236456	DQ246945	Treefish	USA	^28^
	NRRL 32821	DQ094587	DQ236629	DQ247128	Turtle egg	USA	^28^

The original Table 2 and accompanying legend appear below.

The original Article has been corrected.

