# Supplementary material for: Fusarium species isolated from post-hatchling loggerhead sea turtles (Caretta caretta) in South Africa
Source: Sci Rep. 2022 Apr 7;12:5874. doi: 10.1038/s41598-022-06840-1 (PMC8991248; doi:10.1038/s41598-022-06840-1)
Supplement: Supplementary file 1 — Supplementary Legends. [file 41598_2022_6840_MOESM1_ESM.docx]

ONLINE RESOURCE 1: Line drawings of a) macro- and b) microconidia. Illustrating how measurements for conidia were done, i) length of macroconidia, ii) width of macroconidia, iii) length of microconidia and iv) width of microconidia

ONLINE RESOURCE 2: Epidermal scrapes taken from lesions of affected turtles examined by light microscopy (20 to 50 x objective). © Dr. Georgina Cole
