# Supplementary figures and images for: Fusarium species isolated from post-hatchling loggerhead sea turtles (Caretta caretta) in South Africa
Source: Sci Rep. 2022 Apr 7;12:5874. doi: 10.1038/s41598-022-06840-1 (PMC8991248; doi:10.1038/s41598-022-06840-1)

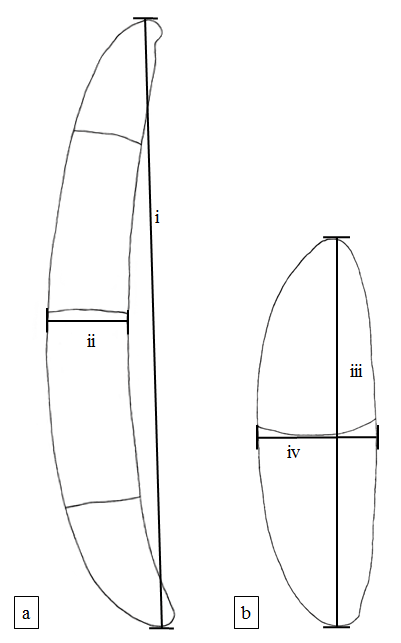

Supplement: Supplementary file 2 — Supplementary Figure 1. [file 41598_2022_6840_MOESM2_ESM.tif]

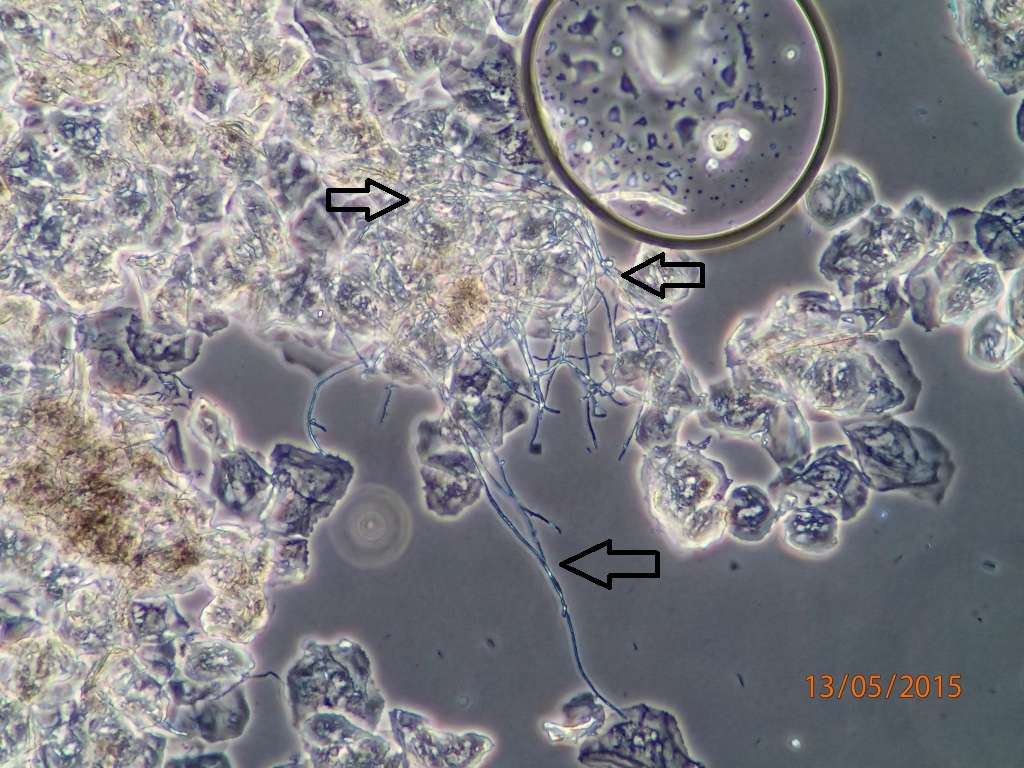

Supplement: Supplementary file 3 — Supplementary Figure 2. [file 41598_2022_6840_MOESM3_ESM.tif]
